# Using Geostatistical Gaussian Simulation for Designing and Interpreting Soil Surface Magnetic Susceptibility Measurements

**DOI:** 10.3390/ijerph16183497

**Published:** 2019-09-19

**Authors:** Piotr Fabijańczyk, Jarosław Zawadzki

**Affiliations:** Warsaw University of Technology, Faculty of Building Services, Hydro and Environmental Engineering, Nowowiejska 20, 00-661 Warszawa, Poland; piotr.fabijanczyk@pw.edu.pl

**Keywords:** industrial areas, soil magnetometry, soil pollution, uncertainty, spatial variability, geostatistical Gaussian simulation, environment

## Abstract

This paper presents a new approach to the assessment of the uncertainty of using geostatistical Gaussian simulation in soil magnetometry. In the study area, numerous measurements of soil magnetic susceptibility were made, and spatial distributions of soil magnetic susceptibility were simulated. The parameters of variograms of soil magnetic susceptibility measured in the study area were determined and compared with those of simulated soil magnetic susceptibility. Regardless of the measurement scheme used, reproducibility of the original semivariograms of soil magnetic susceptibility was satisfactorily achieved when applying simulated values. A nugget effect, a sill, and a range of correlations of variograms of simulated values of soil magnetic susceptibility were similar to those of measured values. When the input data for the geostatistical simulation were averaged, the measured values of soil magnetic susceptibility and simulated spatial distributions were characterized by slightly lower standard deviations in comparison with the result of simulations based on the non-averaged, measured ones. At the same time, however, local variability of soil magnetic susceptibility was reproduced less. The accuracy of the calculations of point parameters and spatial distributions—based on the averaged values of soil magnetic susceptibility—were satisfactory, but when using geostatistical methods, it is recommended to use non-averaged magnetic susceptibility measurements.

## 1. Introduction

Soil magnetometry has frequently been used to detect and determine the potential soil pollution with Potentially Toxic Elements (PTE) emitted by industry, transportation, agriculture, households, and other types of anthropogenic sources. Due to the presence of magnetic particles in anthropogenic pollutants, soil magnetometry has been proposed as a fast and cheap alternative to expensive and time-consuming geochemical laboratory analyses. Numerous studies confirmed statistically significant correlation between industrial pollution and soil magnetic susceptibility [1,2,3,4].

Soil magnetometry is considered as one of the most innovative and promising methods in environmental research [5,6]. Recently, soil magnetometry was a subject of intense development as a method for the screening of soil polluted with toxic elements. Numerous studies were conducted [7,8,9,10] where the methodology of field and laboratory measurements was described and analyzed in detail. However, in addition to the measurement stage, the appropriate analysis of the measured values of magnetic susceptibility—leading to the accurate delineation of spatial distributions and the size of the potentially contaminated area—is an essential procedural requirement. When assessing the degree of soil contamination using soil magnetometry, various types of errors may occur. The first group of measurement errors is related to the apparatus used, the method of soil sampling, and the field measurements technique. These aspects were studied extensively, and numerous scientific papers have been published on this topic [11,12]. However, the biggest challenge is still the determination of the level of soil pollution in places where measurements were not taken, with the simultaneous assessment of the errors of these estimates. These errors may result from difficulties in proper assessment of the spatial variability of soil magnetic susceptibility as well as from other specific properties of the interpolation method [13,14]. Even the most advanced interpolation methods are characterized by the possibility of underestimating or overestimating the interpolated value. Therefore, it is important to estimate the uncertainty of estimated spatial distributions, as it may have a very significant impact on subsequent decisions regarding the classification of certain areas as contaminated or clean.

Simple interpolation methods do not allow the determination of the uncertainty of interpolated values. In the case of geostatistical methods, such as different types of kriging, it is possible to determine a spatial distribution of errors using kriging variance calculations [15]. Such distribution already allows for some estimation of the uncertainty of the interpolated values of magnetic susceptibility of soil and related phenomena. The kriging variance, however, has some limitations arising from problems with assessing the proper model of spatial correlations of phenomena under study.

In this aspect, geostatistical simulations offer an advantage over estimations based on kriging. Geostatistical simulations are better at representing local variability because the small-scale variability is added back into the calculated spatial distributions [16,17,18,19,20]. The averaging of simulated values from geostatistical simulations results in the kriging prediction because the variability added to the predicted value has a mean of zero. After calculating the number of realizations using Sequential Gaussian Simulations (SGS), it is possible to calculate numerous statistics of simulated values at each location within a study area or even calculate histograms or box-and-whisker plots.

Moreover, kriging is based on a local average of measured values and tends to produce smoothed spatial distributions where high-value areas are typically underestimated, while low-value areas are usually overestimated. Geostatistical simulations are better when reproducing high and low values. In soil magnetometry, it can be very important, especially in areas that are classified as hotspots where high values of soil magnetic susceptibility were measured.

This paper presents the analysis of a new approach to the assessment of uncertainty in soil magnetometry using geostatistical Gaussian simulation. More specifically, two of the most common schemes of field measurement of soil magnetic susceptibility were compared in terms of their magnitude of errors of the estimated spatial distributions. It is important to notice that the intention of this paper was not to introduce any new geostatistical modeling methods or updates to an existing one. The goal of this paper was to propose the use of a well-known method of geostatistical Gaussian simulation to be widely used in soil magnetometry studies of soil pollution. The goal was to show the benefits and advantages of using geostatistical simulation, specifically in the soil magnetometry method. This was dictated by the fact that, despite the significant development of soil magnetometry, most of the work focused on the advance in measurement and analytical methods. A very important part of the analysis of the results of field measurements has been neglected to some extent.

In the selected study area, a large number of measurements of soil magnetic susceptibility was made, and several tens of spatial distributions of soil, κ, were simulated and later used to calculate the mean, median, minimum, and maximum of these realizations. As a measure of uncertainty of the simulated values of soil magnetic susceptibility, we have presented spatial distributions of the coefficient of variation of simulated κ, calculated as the standard deviation of simulated values of soil magnetic susceptibility divided by means of simulated values of κ.

Next, simulated and measured values of κ were compared using classic statistics as well as analysis of spatial correlations. The parameters of spatial correlations of κ measured in the study area were determined and compared with those calculated using simulated maps of average κ. The goal of this part of the analysis was to determine which of the data sets allows results that are characterized by parameters of spatial correlation as close as possible to those characterizing the measured κ values.

## 2. Materials and Methods

### 2.1. Study Area

**Figure 1:**
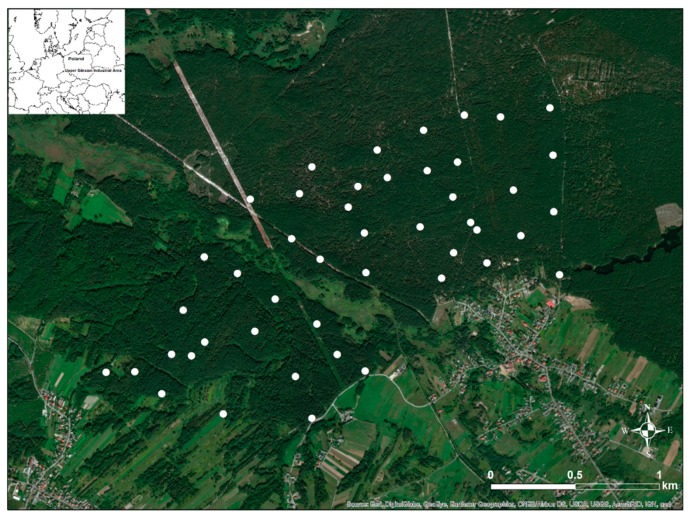
Location of the study area and location of sample points (marked by white dots).

The study area of about 4 km^2^ was located in a forest in Upper Silesian Industrial Area, in southern Poland (Figure 1), WGS84 coordinates 50.323N, 19.450E. The majority of the natural forest where measurements were made was overgrown by coniferous trees. The major types of geological substrata in the study area were sands and gravels, eolian sands, and partially loess, mostly in the south-western part of the area [21].

The surroundings of the analyzed area were characterized by rather complex land management: eastern and north-eastern parts were mostly covered with forests, while the southern and western part were mostly occupied by open, sparsely built-up area. In the past, the surroundings of the study area were intensively used for Pb and Zn ore exploration. At present, only one mining and metallurgical complex is still active and is located about 5 km to the south-east.

### 2.2. Measurements of Soil Magnetic Susceptibility

Magnetic susceptibility was measured on the soil surface using the Bartington MS2D sensor [22]. During field measurements, volume magnetic susceptibility κ was measured and expressed in [10^−5^ SI] units. At the selected location, 10 to 15 single readings were made on the soil surface in a circle with a radius of 2 m. These values of soil magnetic susceptibility measured at every single reading will be furthermore labeled as κ_non-avg_. The total number of κ_non-avg_ values was equal to 460. Within each of the measuring points, values of κ_non-avg_ were averaged, and the calculated average value of soil magnetic susceptibility was furthermore labeled as κ_avg_. The total number of κ_avg_ values was equal to 46. Each of these MS2D readings was assigned with a geographical coordinate.

Firstly, distributions of 10 readings of κ_non-avg_ were analyzed individually at each of 46 sample points in order to determine outlier values, which were defined as lower than Q_25%_ − 1.5 × IQR or higher than Q_75%_ + 1.5 × IQR. After the outlier values of soil magnetic susceptibility were removed, the average magnetic susceptibility was calculated for each of 46 sample points. These averages will be referred to below as κ_avg_. In the final results, two data sets were obtained:

1. the set of 450 κ_non-avg_, non-averaged readings of the MS2D meter performed in each of the 46 measured points,
2. the set of 46 κ_avg_ susceptibility values created after averaging the readings of the MS2D meter which were performed in each of the 46 measured points.

The values of κ_avg_ and κ_non-avg_ were later used as input data in the geostatistical analyses of spatial correlations and simulations.

### 2.3. Spatial Simulations

Spatial distributions of soil magnetic susceptibility were simulated using conditional SGS [16,17,18,19,20]. This type of simulation was selected in order to achieve the best possible replication of the mean, variance, and semivariogram of the measured values of κ_non-avg_ and κ_avg_.

The first step in SGS was to create a grid with a cell size equal to 10 m. Next, following a pre-defined random path, the normal distribution—from where the value of κ was sampled—was centered in the kriging estimates of κ, which were calculated using a covariance model of measured values of κ_non-avg_ or κ_avg_. The average values of all realizations of SGS at sample point locations were approximately equal to measured values of κ_non-avg_ or κ_avg_ depending on which data were used as an input for SGS. The small differences between measured and simulated values could be observed because values were simulated at a grid cell that might not be located exactly in the same place as the sample points. Only one value of κ per location was used in the SGE. In case of the κ_avg_ set, ten values of κ measured per sample point, as well as the XY coordinates of these measurements, were averaged. Then, the average values of κ and XY coordinates were used in simulations. In the case of the simulations based on the κ_non-avg_ set, all 10 values of κ measured per sample point (and their coordinates) were used separately. As a consequence, when ten κ_non-avg_ set was used, many more pairs of κ values existed for each variogram lag. (The number of pairs between measurements is proportional to the square of the number of measurements). A hundred simulations were made separately for two cases when the input data were κ_non-avg_ and κ_avg_ values. These SGS realizations were later used to calculate spatial distributions of:

1. κ_sim-avg_—the average of all simulated realizations,
2. κ_sim-min_—the minimum of all simulated realizations,
3. κ_sim-max_—the maximum of all simulated realizations,
4. κ_sim-std_—the standard deviations of all simulated realizations.

Before running SGS, all input data were tested for normality of their distributions. A Shapiro–Wilk test was selected to check the normality of the input data. The null hypothesis in this test assumes that the analyzed sample comes from a normal distribution. This test was selected because it is the preferred test for normality of the distribution due to its power compared to other alternative tests.

### 2.4. Analyses of Spatial Correlations

Experimental variograms were calculated accordingly to a common formula [15]:

(1) $$\gamma \left(h\right)=\frac{1}{2N}\overset{N}{\underset{i=1}{\sum }}{\left[Z\left(x_{i}\right)-Z\left(x_{i}+h\right)\right]}^{2}$$

where: ***x**_i_* is a location, ***h*** is a lag vector, *Z*(***x****_i_*) is the measured value at location ***x****_i_*, and *N* is the number of pairs spaced by ***h*** vector.

In this work, experimental variograms were calculated based on two different types of data. Firstly, variograms were calculated using values of κ_non-avg_ and κ_avg_ measured at the sample points. Next, variograms were also calculated using simulated values of κ_sim-avg_ separately for two simulation cases where the input was κ_non-avg_ or κ_avg_ values. These simulated values were available not at the measuring points, but in each cell of the simulated grid. The cell size of this grid was equal to 10 m. All experimental variograms were later modeled using the spherical model with a nugget effect. Nugget effect describes the variability between values for distances shorter than a sampling distance and is also related to the variability resulting from measurement and instrumental errors.

## 3. Results and Discussion

Before running the SGS, both data sets of measured values, κ_non-avg_ and κ_avg_, were tested for normality of their distributions. A Shapiro–Wilk test was performed with a significance level of 0.05. The results of this test suggested that for both sets of κ_non-avg_ and κ_avg_, it can be assumed that their distributions are close to normal distribution. On the basis of these results, it was concluded that the κ_non-avg_ and κ_avg_ values could be used in further analyses, variogram calculation, and SGS simulation without data transformation. 

As can be noticed in Table 1, the data set that included all, non-averaged values, κ_non-avg_, was much more frequent than the set of κ_avg_. The average susceptibility values were similar for both sets of data and approximately equal to 65 × 10^−5^ SI. More pronounced differences were observed in the case of quartiles and standard deviation values, where the averaging data resulted in a visible reduction in the spread of susceptibility values. For the κ_avg_, the minimum and maximum susceptibility values were 31 × 10^−5^ SI and 108 × 10^−5^ SI, respectively, and for the set of κ_non-avg_, 14 × 10^−5^ SI and 149 × 10^−5^ SI, respectively.

**Figure 2:**
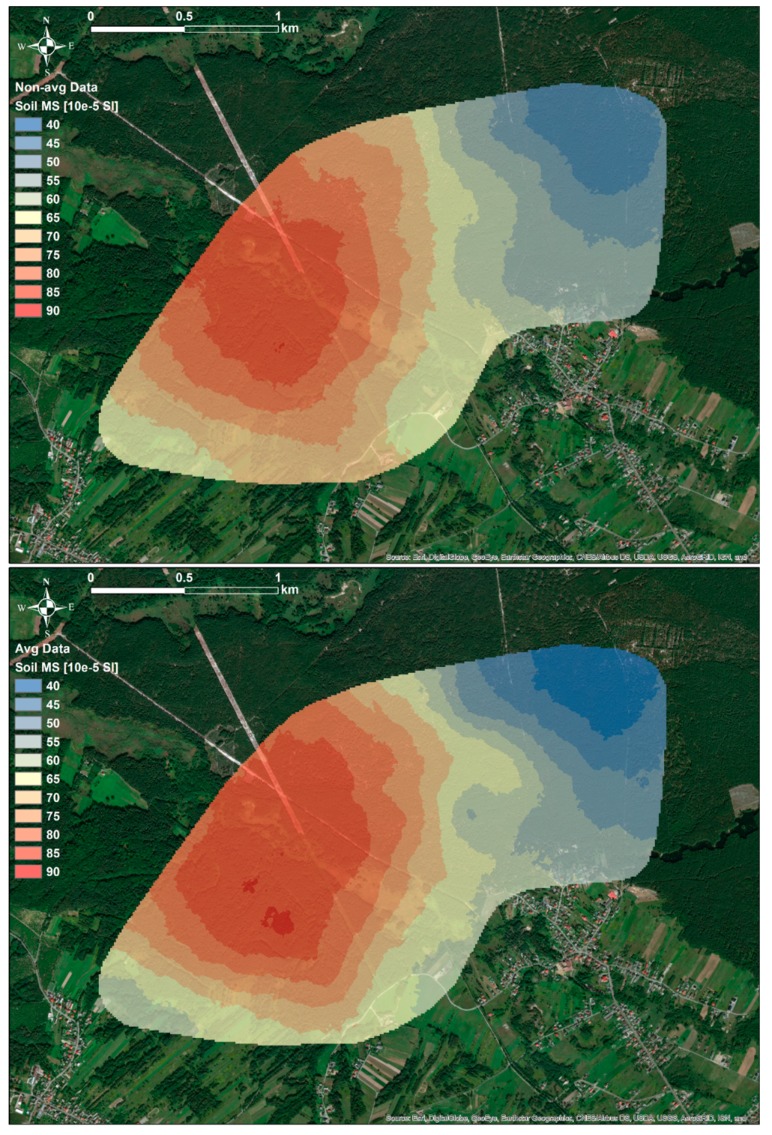
Spatial distributions of κ_sim-avg_ simulated using κ_non-avg_ (upper figure) and κ_avg_ (bottom figure) data sets.

**Figure 3:**
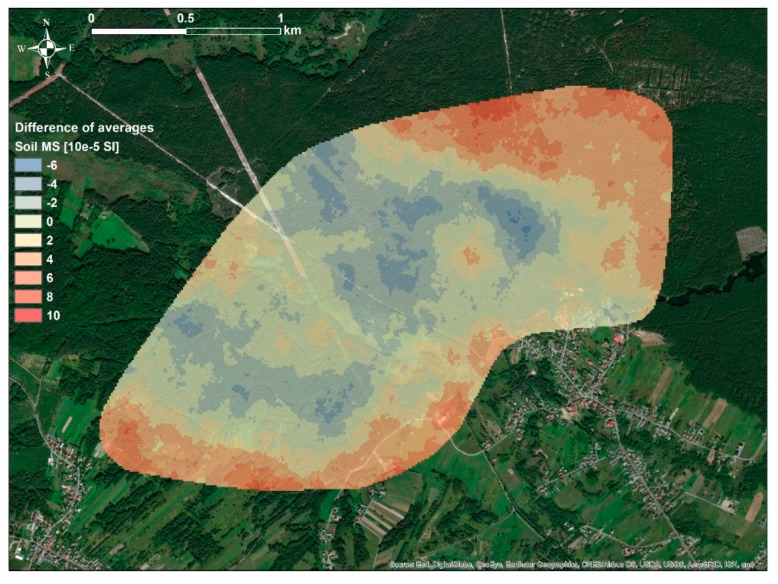
Spatial distribution of differences between κ_sim-avg_ simulated using κ_non-avg_ and κ_sim-avg_ simulated using κ_avg_.

As can be observed in the Figure 2, spatial distributions of κ_sim-avg_ simulated on the basis of two sets of data, κ_non-avg_ and κ_avg_, did not differ significantly. Subareas with high and low values of κ_sim-avg_ were located in similar parts of the study area; in the Western and Eastern parts, respectively. Further analysis of the differences between these distributions, using differential map (Figure 3) between κ_sim-avg_ simulated using κ_non-avg_ and κ_sim-avg_ simulated using κ_avg_ showed that maximum variation between these distributions was in the range of 10 × 10^−5^ SI. This was rather low values in comparison to the range of measured κ_non-avg_ and κ_avg_ values, which was over 100 × 10^−5^ SI. Based on this observation, it might be assumed that the differences between simulated distributions of κ_sim-avg_, which were simulated using data sets κ_non-avg_ and κ_avg_, could be attributed to the specific sampling methodology. 

**Figure 4:**
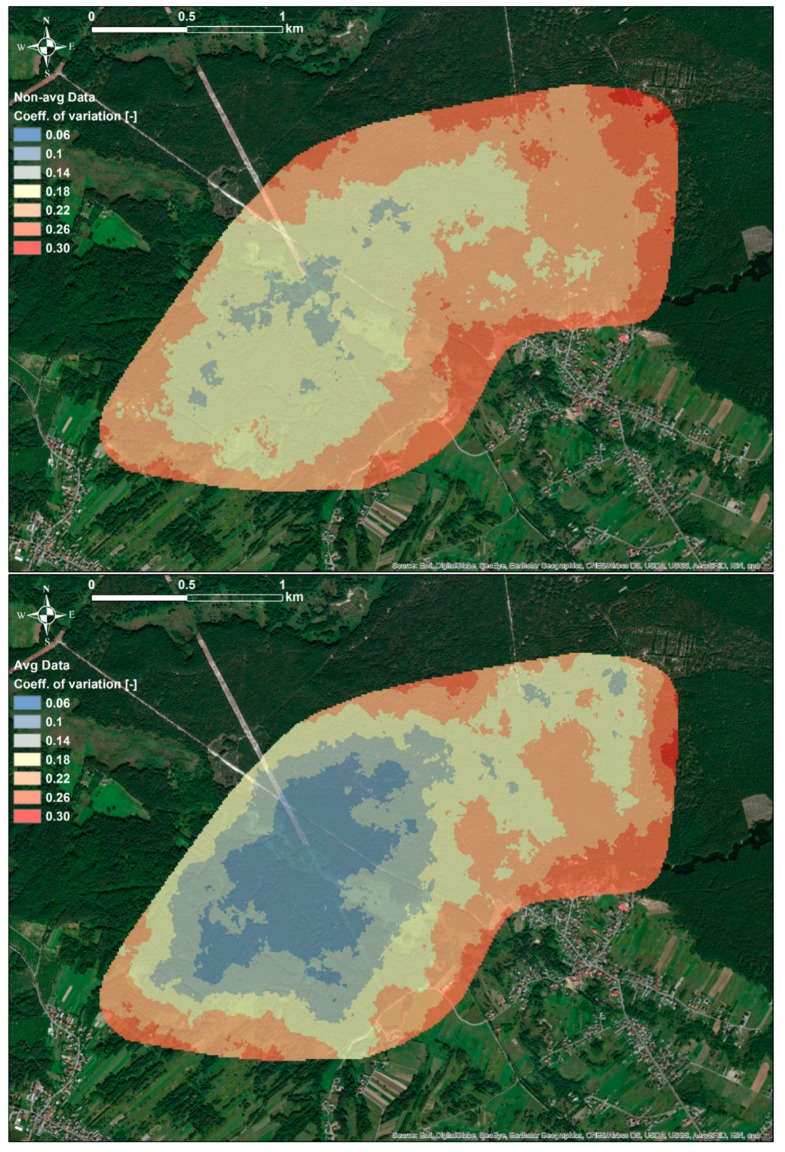
Spatial distributions of the coefficient of variation calculated as κ_sim-std_ divided by κ_sim-avg_, simulated using κ_non-avg_ (upper figure) and κ_avg_ (bottom figure) data sets.

**Figure 5:**
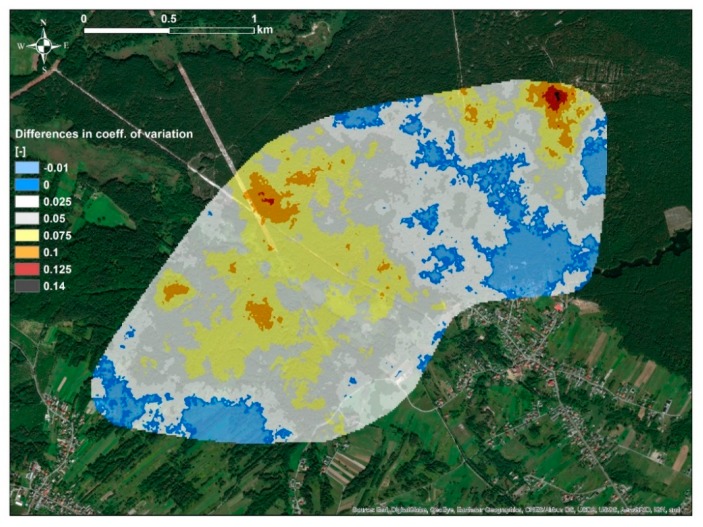
Spatial distribution of differences between coefficients of variation calculated using κ_non-avg_ and κ_avg_ data sets.

Application of SGS made it possible to calculate, at each grid point, numerous realizations of κ values that have similar histograms and variograms as the input data, i.e., κ_non-avg_ and κ_avg_ data sets. Using these realizations, it was possible to assess the variability of the simulated values by calculating κ_sim-avg_, κ_sim-min_, κ_sim-max_, and κ_sim-std_. Firstly, standard deviations of simulated values κ_sim-std_ were calculated, and subsequently, the coefficients of variations, i.e., κ_sim-std_ divided by κ_sim-avg_. As can be observed in Figure 4 and Figure 5, values of the coefficient of variation of κ were under 20% for the majority of the study area. It was also observed that slightly lower values of the coefficient of variation were observed for values simulated on the basis of the κ_avg_ data set. Such observations were due to the fact that the κ_avg_ data set was characterized by lower variability, which was reduced during the averaging of κ_non-avg_ values. As it was analyzed, distributions of measured susceptibility values, κ_non-avg_, at sample points were characterized, in average, by standard deviation, and coefficient of variation equal to 18 × 10^−5^ SI and 27%, respectively. Therefore, it was evident that the variability of simulated values of soil magnetic susceptibility at individual points was at a similar level to that of measured values.

**Figure 6:**
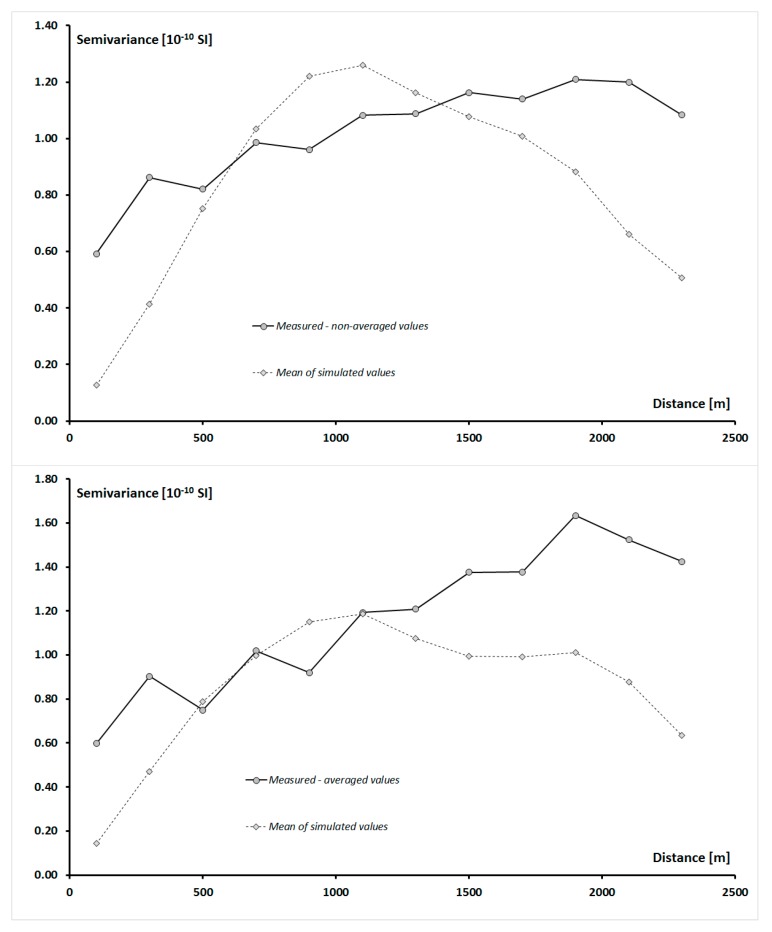
Experimental variograms of measured and simulated values of soil magnetic susceptibility using κ_non-avg_ data (upper figure) and κ_avg_ (bottom figure).

In the next step, the characteristics of spatial correlations of κ were analyzed using measured κ_avg_ and κ_non-avg_ data sets, as well as simulated values of κ_sim-avg_, κ_sim-min_, κ_sim-max_, and κ_sim-std_. For this purpose, experimental variograms (Figure 6) were calculated with 12 lags, and a lag distance equal to 200 m. Before the calculation of experimental variograms, input data were transformed using the normal-score transformation. After the experimental variograms of measured and simulated soil magnetic susceptibility were calculated, they were modeled using a spherical model with the nugget effect. The goal of this part of analysis was to investigate if the parameters of spatial correlations of simulated κ_sim-avg_, κ_sim-min_, κ_sim-max_, and κ_sim-std_ were similar to those of measured κ_non-avg_ values.

As can be observed in Figure 6, experimental variograms of simulated κ_sim-avg_ had a similar shape to the variograms of measured κ_non-avg_ and κ_avg_ values, especially where they achieved sill. In order to investigate the similarities between spatial correlations of κ_sim-avg_, κ_non-avg_, and κ_avg_ more precisely, all variograms were modeled, and the parameters of these models were placed in Table 2.

Comparison of the parameters of spherical models of measured and simulated values of soil magnetic susceptibility showed that the correlation range of κ_avg_ variogram was noticeable but not much longer than the correlation range of κ_non-avg_ variogram. As for the modeled nugget effect, it was lower for κ_avg_ variograms in comparison with κ_non-avg_ ones. Such observation could be explained by the fact that during the averaging of values of soil magnetic susceptibility, the impact of outliers was reduced. As can be observed in Table 2, the sill of the spherical model of κ_avg_ was slightly higher as the sill of κ_non-avg_. Referring these observed differences to the calculated experimental variograms, it can be noted that the differences in the sill values of spherical models of κ_non-avg_ and κ_avg_ concerned practically only distances above 1700 m. This distance was longer than the ranges of correlation of both κ_non-avg_ and κ_avg_ values. Differences in sill values could result mainly from a very large difference in the number of values of κ_non-avg_ and κ_avg_, which were equal to 450 and 46, respectively. As a result, according to the Formula (1), on the basis of which the semivariance values were calculated, in the case of a variogram of κ_non-avg_ values, it was possible to find many more pairs of sample points. Due to the fact that the semivariance values of κ_non-avg_ were calculated on the basis of a much larger number of pairs of points than in the case κ_avg_, the sill value of the spherical model of κ_non-avg_ was lower. However, it should be stated that the spatial characteristics of measured κ_non-avg_ and κ_avg_ values were rather similar, and the observed differences resulted from the sampling method. 

In the next stage, the parameters of the variograms of measured κ_non-avg_, κ_avg_, and simulated κ_sim-avg_ values were compared. In each case, the variogram determined from the measured κ_non-avg_ or κ_avg_ values was used as the reference point. As can be noticed in Table 2, the comparison was made separately for values simulated when the input data for SGS was set κ_non-avg_ and κ_avg_.

As it was observed, values of nugget effect of variograms of simulated values κ_sim-avg_ were significantly lower than that of variograms of measured κ_non-avg_ and κ_avg_. Simultaneously, the comparison of the parameters of spherical models showed that variograms of simulated values, κ_sim-avg_, were characterized by lower values of nugget effect than variograms of measured values of κ_non-avg_ and κ_avg_ data. Such observations might suggest that SGS was quite effective in recreating the local variability of soil magnetic susceptibility, especially for distances shorter than a distance between sample points. Measured values of soil magnetic susceptibility were not available for such small distances, though they were available for simulated data sets, κ_sim-avg_ values were simulated for each simulation grid cell with a size of 10 m.

Sill values of variogram models of κ_sim-avg_ simulated using κ_non-avg_ and κ_avg_ data sets were comparable. It is important to underline here that a ratio of nugget effect to sill, which ranges from 0 to 1, is often recognized as a critical measure to define the spatial dependence of soil properties [23,24]. The closer this ratio is to zero, the stronger spatial correlations are observed. Precise assessment of this ratio is crucial to the quality of the results of geostatistical analyses in soil magnetometry. Usually, nugget effect is much more difficult to determine than sill. As it was observed, spherical models of simulated κ_sim-avg_ were characterized by a shorter range of correlation in comparison with variograms of measured κ_non-avg_ or κ_avg_. As can be noticed in Table 2, this difference was equal to about a few hundred meters. The explanation of such observations is related to the fact that values of simulated κ_sim-avg_ might be characterized by greater spatial variability at shorter distances. It is related to the fact that simulated data were much more numerous than the measured data and were available at all 60 thousand grid cells, so small-scale spatial variability of magnetic susceptibility was well reproduced.

## 4. Conclusions

The most pronounced differences between spatial distributions of the average soil magnetic susceptibility, simulated using non-averaged and averaged measurements, were found in places with a high and low level of magnetic susceptibility. In the parts of the study area where the lowest magnetic susceptibility was observed, values of soil magnetic susceptibility simulated using non-averaged data were higher than those simulated using averaged data. In the parts of the study area where soil magnetic susceptibility was the highest, the opposite situation was observed. 

The spatial variation of soil magnetic susceptibility was distinctly higher in the case of simulations made on the basis of non-averaged measured data due to the considerable loss of information about the small-scale variability of soil magnetic susceptibility during data averaging. This resulted in the underestimation of the values of a nugget effect when using averaged data which, as a consequence, may lead to incorrect assessment of the level and extent of soil magnetic susceptibility distributions.

For longer distances, regardless of the measurement scheme used, reasonable reproducibility of the original semivariograms of soil magnetic susceptibility was achieved by simulated values. Variograms of the average of simulated values of soil magnetic susceptibility had similar sills and ranges of correlation to those of the variogram calculated from values measured in the study area. However, as before, the local variability of soil magnetic susceptibility was better reproduced when using non-averaged values than averaged ones, regardless of the fact whether the data is measured or simulated. This result confirms that it is favorable not to average magnetometric measurements for geostatistical analyses.

Thus, our study showed that the geostatistical Gaussian simulation provides deep insight into the variability and precision of soil magnetometry measurements at different distances, allowing for more efficient planning of soil field measurements.

## Figures and Tables

**Table 1:** Descriptive statistics of measured values of κ_non-avg_ and κ_avg_.

	κ_avg_	κ_non-avg_
	(10^−5^ SI)	
**Average**	65.2	65.7
**Q_25%_**	47	46
**Q_75%_**	82	87
**Minimum**	31	14
**Maximum**	108	149
**Standard deviation**	20	27
**Number**	46	450

**Table 2:** Parameters of variogram spherical models measured and simulated κ values.

	Nugget Effect	Sill	Range of Correlation
	(10^−10^ SI)		(m)
**Measured Values**			
**κ_non-avg_**	0.636	1.133	1580
**κ_avg_**	0.520	1.320	1700
**Values Simulated Using κ_non-avg_**			
**κ_sim-avg_**	0	1.220	1100
**Values Simulated Using κ_avg_**			
**κ_sim-avg_**	0	1.190	1150
